# The identification and validation of histone acetylation-related biomarkers in depression disorder based on bioinformatics and machine learning approaches

**DOI:** 10.3389/fnins.2025.1479616

**Published:** 2025-04-30

**Authors:** Lu Zhang, YuJing Lv, Mengqing Ma, Jile Lv, Jie Chen, Shang Lei, Yi Man, Guimei Xing, Yu Wang

**Affiliations:** ^1^Department of Neurology, the First Affiliated Hospital of Anhui Medical University, Hefei, China; ^2^Department of Neurology, Anhui No. 2 Provincial People’s Hospital, Hefei, China; ^3^Graduate School, Bengbu Medical University, Bengbu, China; ^4^Department of Psychiatry, Affiliated Psychological Hospital of Anhui Medical University, Hefei, China; ^5^Department of Oncology, Anhui Jimin Cancer Hospital, Hefei, China; ^6^Department of Education, Anhui No. 2 Provincial People’s Hospital, Hefei, China

**Keywords:** depression disorder, histone acetylation, hub genes, immune infiltration, bioinformatics

## Abstract

**Background:**

Some studies indicated that histone modification may be involved in depression disorder (DD). The maintenance of the histone acetylation state is the work of histone acetyltransferase (HAT) and histone deacetylase (HDAC), which is thought to be a potential diagnostic biomarker of depression. However, it is still unknown how histone acetylation-related genes (HAC-RGs) contribute to the onset and progression of DD.

**Methods:**

GSE76826 and GSE98793were obtained from the Gene Expression Omnibus (GEO) database, HAC-RGs were acquired from the GeneCards database. Initially, the differentially expressed genes (DEGs) in GSE76826 were investigated. We used weighted gene co-expression network analysis (WGCNA) to screen key module genes. Candidate genes were selected by intersecting DEGs, key module genes, and HAC-RGs, followed by functional analysis. Two machine learning algorithms were used to identify hub genes, which were used for drug prediction, immunological infiltration studies, nomogram construction, and regulatory network building. The expression levels were verified using the GSE76826 and GSE98793 datasets. Hub gene expression levels in the clinical samples were verified using reverse transcription quantitative PCR (RT-qPCR).

**Results:**

The 23 candidate genes were obtained by intersecting 2,316 DEGs, 1,010 HAC-RGs and 2,617 key module genes. Three hub genes (*JDP2*, *ALOX5*, and *KPNB1*) were gained by two machine learning algorithms. The nomogram constructed based on these three hub genes showed high predictive accuracy. Additionally, the three hub genes were enriched in the kegg_ribosome. The 9 different immune cells were identified in GSE76826, which were associated with three hub genes. A hub gene-drug network (98 nodes, 106 edges) and an lncRNA-miRNA-mRNA network (56 nodes, 87 edges), were built using the database. The expression level verification indicated that, with the exception of the KPNB1 gene, the DD group had higher levels of JDP2 and ALOX5 and that the expression patterns in GSE76826 and GSE98793 were consistent, with RT-qPCR confirming higher ALOX5 and JDP2 expression in DD samples.

**Conclusion:**

This study identified three hub genes (JDP2, ALOX5, and KPNB1) associated with histone acetylation, offering new insight into the diagnosis and treatment of DD.

## 1 Introduction

DD is one of the most common mental illnesses, characterized by persistently depressed mood, cognitive impairment, and, in severe cases, self-harm and suicidal behaviors (Jin et al., 2019). More than 300 million people suffer from DD worldwide, contributing to a substantial global disease burden (GBD 2019 Diseases and Injuries Collaborators, 2020). While pharmacological treatments are the primary intervention for DD, their efficacy is limited, and they often come with numerous adverse effects and place a heavy financial, psychological, and physical burden on patients. Additionally, clinical practice currently lacks objective biomarkers for DD. Studies have revealed that the etiology of DD is multifactorial, predominantly including genetic and environmental variables as well as their interactions. Recent research on epigenetic regulation in DD suggests that this disorder may be associated with abnormal monoamine neurotransmitter secretion, increased oxidative stress, inhibition of neurotrophic factors, excessive secretion of inflammatory cytokines, and activation of the hypothalamic-pituitary-adrenal (HPA) axis (Shadrina et al., 2018; Himmerich et al., 2019; Majd et al., 2020; Wang et al., 2020). Therefore, understanding these intricate biological processes is crucial for developing efficient DD diagnosis and treatment strategies.

Recently, there has been a notable surge of interest in the relationship between DD and epigenetics. Epigenetics serves as a potential link between genetic and environmental influences. Originally proposed by Waddington, epigenetics refers to the regulation of gene expression without altering DNA sequences (Waddington, 1959). The primary mechanisms of epigenetic regulation include DNA methylation, histone post-translational modifications, genomic imprinting, and non-coding RNAs (Portela and Esteller, 2010). Previous research has highlighted the critical role of epigenetic alterations in neurological disorders, including DD, Parkinson’s disease, Alzheimer’s disease, and Huntington’s disease (Shukla and Tekwani, 2020). Among various forms of epigenetic modifications, post-translational modifications of histones have been shown to influence chromosome conformation, thereby affecting gene expression. Epidemiological analyses reveal that although monozygotic twins exhibit similar histone modifications at birth, these differences manifest with age, resulting in varying risks of developing DD (Lockwood et al., 2015). This suggests that epigenetic characteristics may be modified by environmental factors, which increases the susceptibility to DD. Histone modifications, a type of epigenetic alteration, regulate gene expression genes by influencing chromosome structure. The maintenance of histone acetylation state is a function of histone acetyltransferase (HAT) and histone deacetylase (HDAC), which are thought to be potential diagnostic biomarkers of depression. Imbalances in HAT and HDAC activities lead to aberrant histone acetylation, which causes aberrant behavior and reduced cognitive function by compromising synaptic plasticity. Studies have reported that prenatal stress reduces BDNF expression and increases HDAC expression in the hippocampal regions, which affects synaptic and neuronal plasticity. This may be due to the emergence of behaviors resembling those of depression and anxiety. Similarly, adult stress increases hippocampal HDAC5 expression and MeCP2 levels in the BDNF promoter (Park et al., 2021). Furthermore, histone hyper-and hypoacetylation influence physiological balance in neurons and promote the accumulation of pathological proteins (Kabir et al., 2023). However, the specific mechanism by which histone acetylation-related genes contribute to the development of DD has not yet been documented.

In this study, we utilized the least absolute shrinkage and selection operator (LASSO) analysis to screen for genes with diagnostic value for DD, identify differentially expressed histone acetylation-related genes, construct a diagnostic model, and assess its diagnostic performance. In addition, we examined the correlation between these biomarkers and immune cell infiltration to elucidate the role of the immune system in DD onset. A lncRNA-miRNA-mRNA gene network was also constructed. The findings of this study provide a theoretical foundation for the diagnosis and treatment of DD, offering insights into the pathophysiology of the condition.

## 2 Materials and methods

### 2.1 Data source and processing

Training set GSE76826 and Validation set GSE98793 relevant to DD, were obtained from the Gene Expression Omnibus (GEO) database.^1^ GSE76826 contains 10 DD blood samples and 12 control samples. The GSE98793 dataset contained 64 DD blood samples and 64 control samples. A total of 1,010 histone acetylation-related genes (HAC-RGs) (relevance score > 5) were obtained from the GeneCards database.^2^ R-package “limma” (version 3.52.4) was used to obtain the differentially expressed genes (DEGs) in GSE76826 between DD and control samples (|log2FC(fold change)| > 0.5, *p*-value < 0.05) (Wang et al., 2022; Ritchie et al., 2015).

### 2.2 Weighted gene co-expression network analysis

Outlier samples were first identified and removed through clustering. To ensure that gene interactions conformed to a scale-free distribution, we determined the optimal soft threshold for the data. A co-expression module was then constructed using the hybrid dynamic tree-cutting algorithm, with a minimum module size of 100 genes. The module most strongly correlated with the studied traits was identified as the key module. Finally, key module genes were screened through the establishment of suitable thresholds based on gene significance (GS) and module membership (MM) (Wang et al., 2022; Langfelder and Horvath, 2008).

### 2.3 Functional analysis of candidate genes

DEGs, HAC-RGs, and critical module genes were intersected to identify candidate genes. Kyoto Encyclopedia of Genes Genomes (KEGG) and Gene Ontology (GO) studies using the R-package “clusterProfiler” (version 4.4.4) (*p*-value < 0.05). Cellular components (CC), molecular functions (MF), and biological processes (BP) were incorporated into the GO item (Wang et al., 2022; Ao et al., 2023; Zhao et al., 2021). To create a protein-protein interaction (PPI) network of potential genes, we used The STRING database (correlation threshold: 0.15) (Zhang et al., 2022; Chen et al., 2022).

### 2.4 Machine learning screening

Based on candidate genes, (LASSO) (R-package “glmnet,” version 4.1.7) and the Boruta algorithm were utilized to identify feature genes. LASSO regression can yield regression coefficients that are equal to zero, thereby facilitating the construction of an interpretable model. The Boruta algorithm, on the other hand, identifies the most relevant features that correlated with the dependent variable. Hub genes were obtained by intersecting the feature genes identified by the two machine learning algorithms (Zhang et al., 2022; Zhu et al., 2022).

### 2.5 Construction of nomogram

The nomogram containing hub genes were constructed using R-package “rms” (version 6.7.1). Base on training set, the receiver operating characteristic (ROC) curve was drawn using R-package “pROC” (version 1.18.0) to evaluate the model accuracy (Wang et al., 2022; Sun et al., 2022).

### 2.6 Gene set enrichment analysis

According to the correlation (hub genes vs. all genes in GSE76826), and gene set enrichment analysis (GSEA) enrichment analysis was carried out for the hub genes using R-package “clusterProfiler” (version 4.4.4) and org.Hs.eg.db (|NES| > 1 & NOM, *p*-value < 0.05) (Zhang et al., 2022).

### 2.7 Immune analysis

The single-sample gene set enrichment analysis (ssGSEA) algorithm was used to calculate the distribution proportions of the 28 immune cell types in GSE76826. Differences in immune cells were compared using *t*-test (DD *vs* control). Spearman’s correlation was calculated between hub genes and differential immune cells (Zhang et al., 2022; Lv et al., 2022), and the ggcorrplot (v0.1.3) function was used to plot a correlation heatmap of hub genes and differentially expressed immune cells (Tian et al., 2023).

### 2.8 Construction of lncRNA-miRNA-mRNA network

The miRNAs regulating the hub genes were predicted using miRDB^3^ and the miRWalk database.^4^ The predicted miRNAs from the two databases were then intersected to identify common miRNAs. The Starbase database^5^ was used to predict the lncRNAs (clipExpNum > 20) that corresponded to the miRNAs. The Cytoscape software was used to visualize the lncRNA-miRNA-mRNA network (Shannon et al., 2003; Li et al., 2022).

### 2.9 Drug prediction and validation of hub genes

Utilizing the comparative toxicogenomics database (CTD),^6^ Potential medications linked to hub genes were predicted using the Comparative Toxicogenomics Database.^7^ Cytoscape software visualizes the hub gene drugs that inhibit the expression of the hub gene network (Li et al., 2022). In addition, we analyzed the expression of key genes in the training set GSE76826 and the external validation set GSE98793. The ggplot (version 3.4.4) function was used to create violin plots and box plots. The Wilcoxon rank-sum test (non-parametric test) was applied to calculate the significance of inter-group differences, with a threshold of *p* < 0.05 to generate significance symbols.

### 2.10 Statistical analysis

Hub gene expression was examined using the R package “ggplot.” This study’s analysis was performed using the R programming language. Tests for group differences were conducted using either the Wilcoxon rank-sum test or *t*-test. Statistical significance was set at *p* < 0.05 significant.

### 2.11 Experimental validation by reverse transcription quantitative PCR

TRIzol (Ambion) was used to extract total RNA from the blood specimens. A NanoPhotometer N50 was used to assess RNA concentration and purity. The SureScript First Strand cDNA Synthesis Kit (Servicebio) was used for the reverse transcription of total RNA into cDNA. The 2x Universal Blue SYBR Green qPCR Master Mix was used for qPCR analysis. The qPCR reaction was run for 40 cycles, with the following parameters: initial denaturation for 1 min at 95°C, denaturation for 20 s at 95°C, annealing for 20 s at 55°C, and extension for 30 s at 72°C. Table 1 contains a list of primer sequences. The relative expression of hub genes was determined using the 2^−△△Ct^ method, with relative expression levels normalized to the endogenous reference GAPDH. And we used the ggplot function for plotting, with the geom_bar function to create bar plots. The *t*-test was applied to calculate the *p*-value between groups and generate significance symbols.

**Table 1:** The sequences of the primers for qPCR.

Gene name	Primer sequences
ALOX5 F	CCAGACCATCACCCACCTTC
ALOX5 R	CCTTGTCAAAGAGGCCACAC
JDP2 F	CTCTCAGTCTTGGGGCCTTC
JDP2 R	CCAGGCATCATAGCAGGAGG
KPNB1 F	CCCATTTGGAGGGAGGAAGTA
KPNB1 R	GTTCCACAAGGAAAGTGGGC
GAPDH F	CGAAGGTGGAGTCAACGGATTT
GAPDH R	ATGGGTGGAATCATATTGGAAC

## 3 Results

### 3.1 The 23 candidate genes were obtained

**Figure 1:**
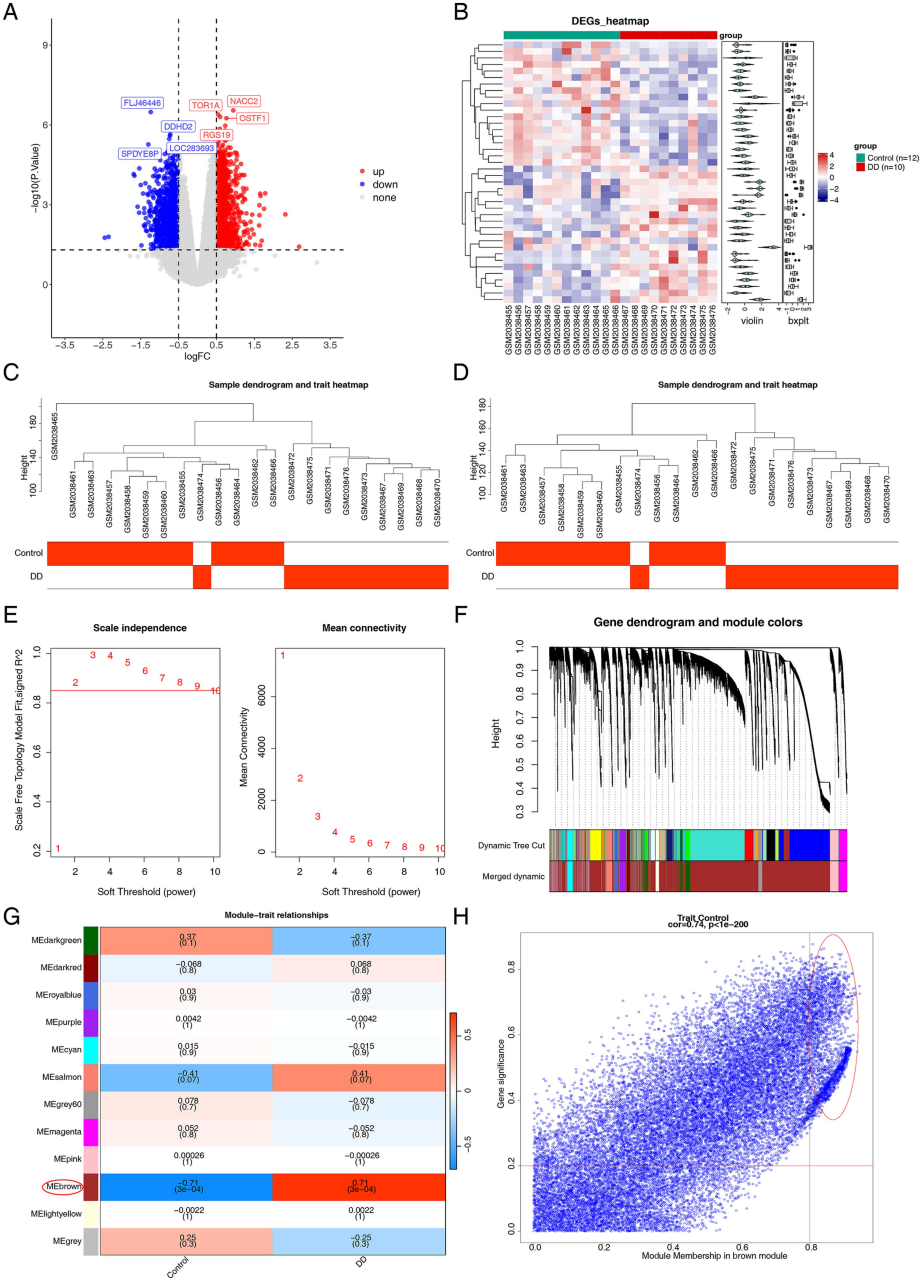
DEGs identification in peripheral serum from depressed patients. **(A)** Volcano plot of GSE76826. Red and blue represent upregulated and downregulated DEGs, respectively, and gray represents no difference. **(B)** Heatmap of GSE76826. **(C,D)** Sample clustering plots after removing outlier samples. **(E)** Examination of the mean connectivity and scale-free index for different soft-thresholding powers. **(F)** Clustering dendrogram for the development of similar modules. Each color represents a module. **(G)** Relations between modules and traits. In the heatmap, a clinical characteristic is represented by each column and a ME by each row. The *p*-value and correlation coefficient for each cell are displayed. **(H)** A scatter plot showing how the MEbrown module’s gene significance (GS) and module membership (MM) relate to one another. Solid dots are utilized to indicate genes having MM ≥ 0.5 and GS ≥ 0.5.

To identify differentially expressed genes between the normal group and the DD group, the results of the differential expression analysis showed, in GSE76826 (DD vs. control), we acquired 2,316 DEGs, of which 1,298 were up-regulated and 1,018 were down-regulated (Figures 1A,B). To identify the gene modules most closely related to DD, we performed weighted gene co-expression network analysis (WGCNA) analysis. The sample clustering diagram after removing the outlier sample is presented in Figures 1C,D. When β = 8 and *R*^2^ = 0.85, the mean connectivity converges to 0 (Figure 1E). After clustering similar modules, 12 co-expression modules were obtained (threshold: 0.8) (Figure 1F). The results of the correlation study indicated that the MEbrown module had the strongest significant positive correlation with DD (*R*^2^ = 0.71, *p* = 3e-04). The MEbrown module, which contained 23,248 genes, was identified as crucial (Figure 1G).

**Figure 2:**
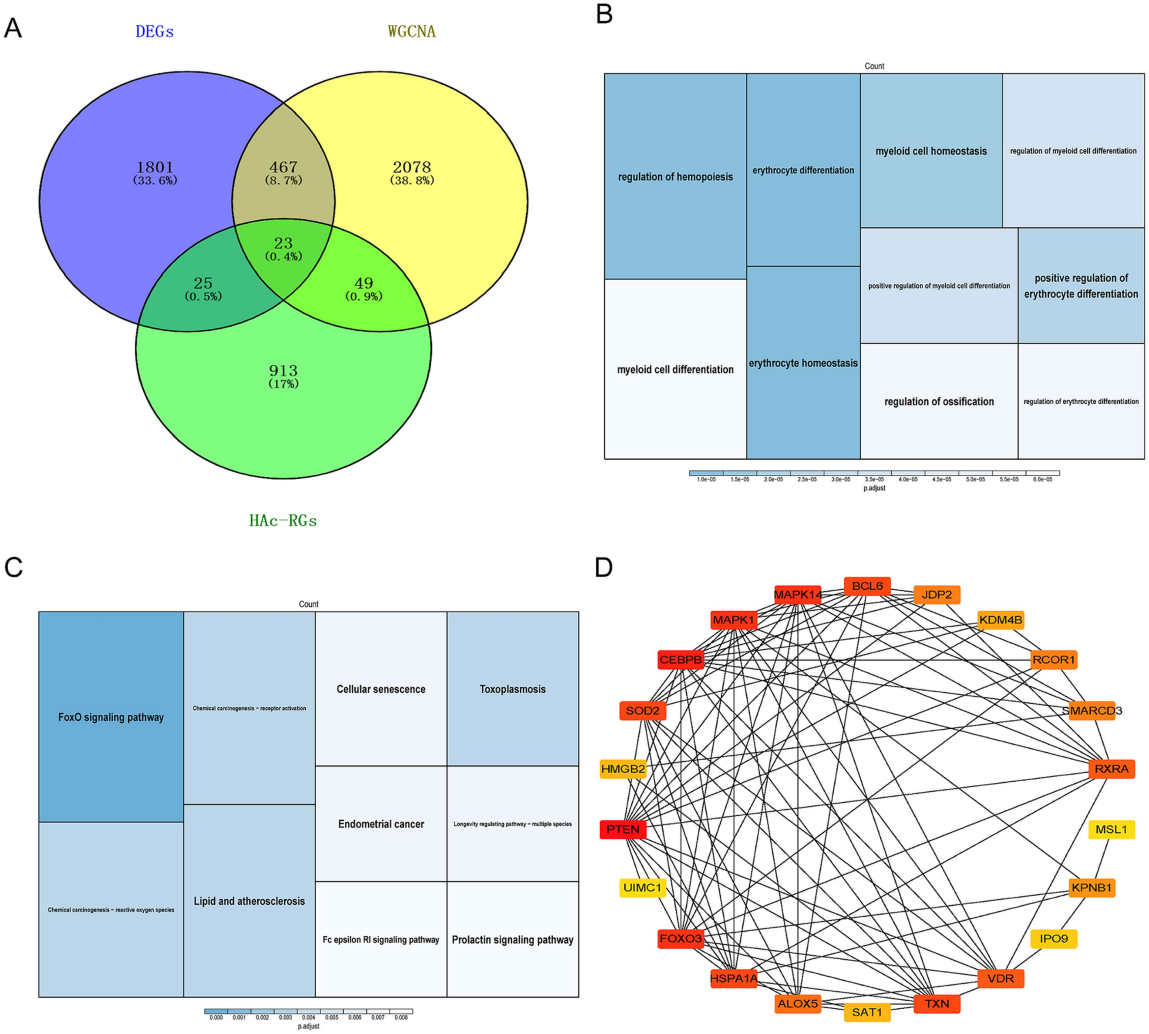
Candidate gene functional analysis. **(A)** Venn diagram showing the overlap between DEG, HAC-RG, and key module genes. **(B)** GO enrichment analysis, **(C)** KEGG enrichment analysis. **(D)** Interaction between MAPK1 and MAPK14, CEBPB, and SOD2, etc. The deeper the red color in the image, the higher the interaction level.

Further screening of 2,617 important module genes was conducted based on MM > 0.8 and GS > 0.2 (Figure 1H). By combining DEGs, HAC-RGs, and key module genes, 23 candidate genes were identified (Figure 2A). To explore the common functions and related pathways of the candidate genes, we performed enrichment analysis, and the results of the GO analysis showed, the candidate genes in BP were primarily related to erythrocyte differentiation. In CC, candidate genes were enriched in the RNA polymerase II transcription regulator complex. In the MF, the candidate genes were associated with DNA-binding transcription factor binding (Figure 2B). In the KEGG pathways, the candidate genes were mainly concentrated in the FoxO signaling pathway (Figure 2C). Based on the 23 candidate genes, the PPI network contained 22-nodes and 89 edges. Notably, strong interaction was observed between MAPK1 and MAPK14, CEBPB, and SOD2 (Figure 2D).

### 3.2 Three hub genes were identified by machine learning

**Figure 3:**
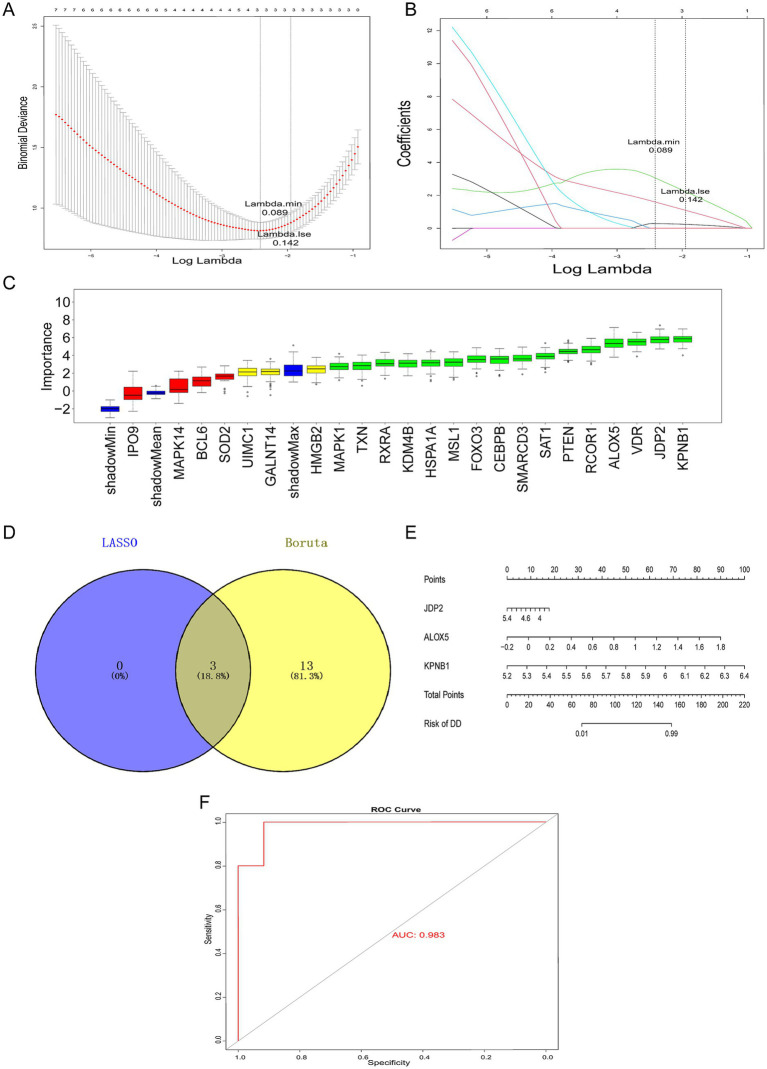
Construction and validation of diagnostic histone acetylation-related gene signatures for DD. Screening diagnostic markers for DD using a comprehensive approach. **(A,B)** LASSO regression analysis used to identify hub genes. **(C)** Boruta algorithm analysis. **(D)** Hub genes. **(E)** Nomogram of hub genes. **(F)** ROC curve diagnostic performance of hub genes.

To further screen for key genes that could serve as diagnostic biomarkers for DD, we performed machine learning-based analysis on the candidate genes. Three characteristic genes were obtained using LASSO algorithm: *JDP2*, *ALOX5* and *KPNB1* (Figures 3A,B). Following analysis with Boruta algorithm, 16 characteristic genes were identified: *JDP2*, *ALOX5*, *KPNB1*, *PTEN*, *MSL1*, *RCOR1*, *SAT1*, *HSPA1A*, *TXN*, *RXRA*, *KDM4B*, *MAPK1*, *VDR*, *CEBPB*, *FOXO3*, and *SMARCD3* (Figure 3C). By intersecting the characteristic genes, three hub genes (*JDP2*, *ALOX5,* and *KPNB1*) were identified (Figure 3D). JDP2 was a member of the stress protein family, and it regulated gene expression by interacting with other transcription factors (Yu et al., 2019). ALOX5 was an iron-containing non-heme dioxygenase that regulated cell death through inflammation and lipid peroxidation (Li et al., 2023). KPNB1 was a key protein in the karyopherin beta family and played a critical role in epigenetic regulation (Dong et al., 2018). Moreover, the study discovered that the identified hub genes are functionally associated with the biological pathways depicted in Figures 1, 2. Specifically. JDP2 modulates erythroid differentiation and transcriptional activity by interacting with DNA-binding transcription factors (Liila-Fogarty et al., 2024; Jin et al., 2006). ALOX5 is implicated in the FoxO signaling pathway and contributes to transcriptional regulation (Liu et al., 2024). KPNB1 mediates nuclear import of transcription factors through recognition of DNA-binding domains (Li et al., 2024). As shown in Figure 3E, a nomogram based on these three hub genes was constructed. Moreover, the area under the curve (AUC) values of the model were > 0.9, indicating that the model had an excellent predictive ability (Figure 3F).

### 3.3 JDP2, ALOX5, and KPNB1 were correlated with ribosome pathway

**Figure 4:**
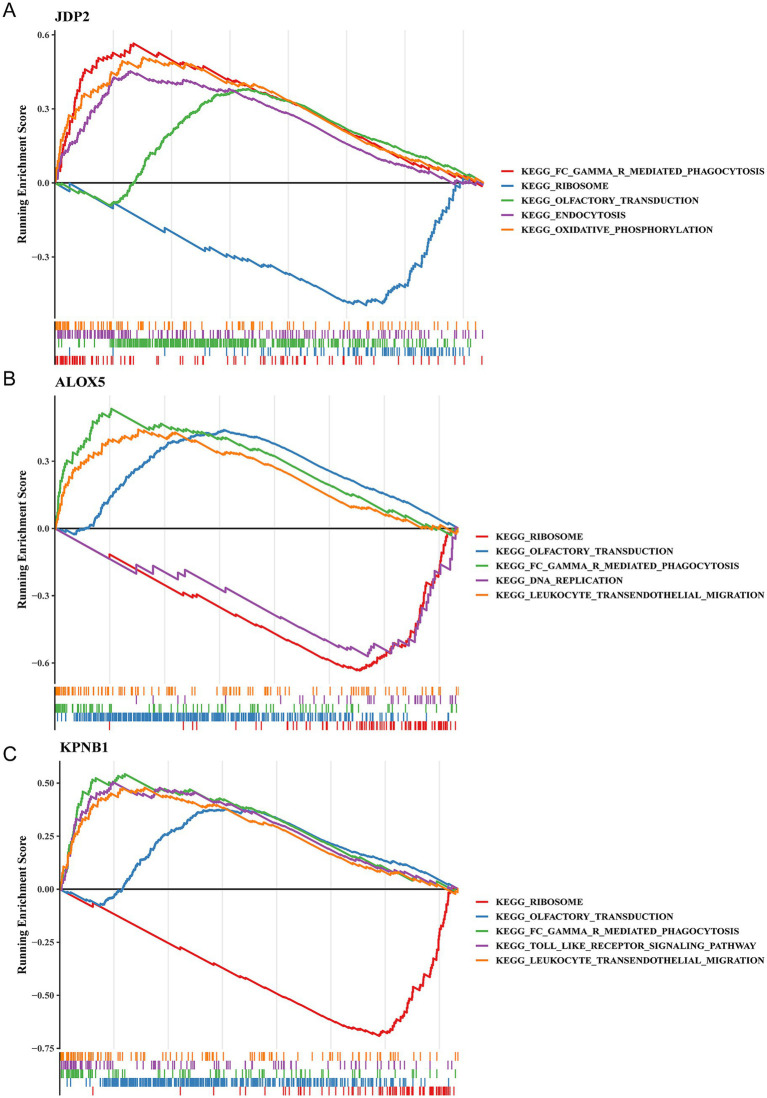
Three hub genes were correlated with ribosome pathway. **(A–C)** GSEA functional analysis of hub genes **(A)** JDP2, **(B)** ALOX5, **(C)** KPNB1.

To further investigate the relevant signaling pathways and potential biological mechanisms involved in key genes. We performed GSEA analysis based on the three hub genes. *JDP2* was enriched in 69 KEGG pathways, including the kegg_fc_gamma _ r-mediated phagocytosis (Figure 4A). *ALOX5* was associated with 52 KEGG pathways, including the kegg_dna_replication (Figure 4B). *KPNB1* was associated with 71 KEGG pathways, including the kegg_toll_like_receptor_signaling_pathway (Figure 4C). Furthermore, the three hub genes were enriched in kegg_ribosome, kegg_fc_gamma_r_ mediated_phagocytosis and kegg_olfactory_transduction, indicating that these genes might have affected DD through these pathways.

### 3.4 Nine different immune cells were associated with three hub genes

**Figure 5:**
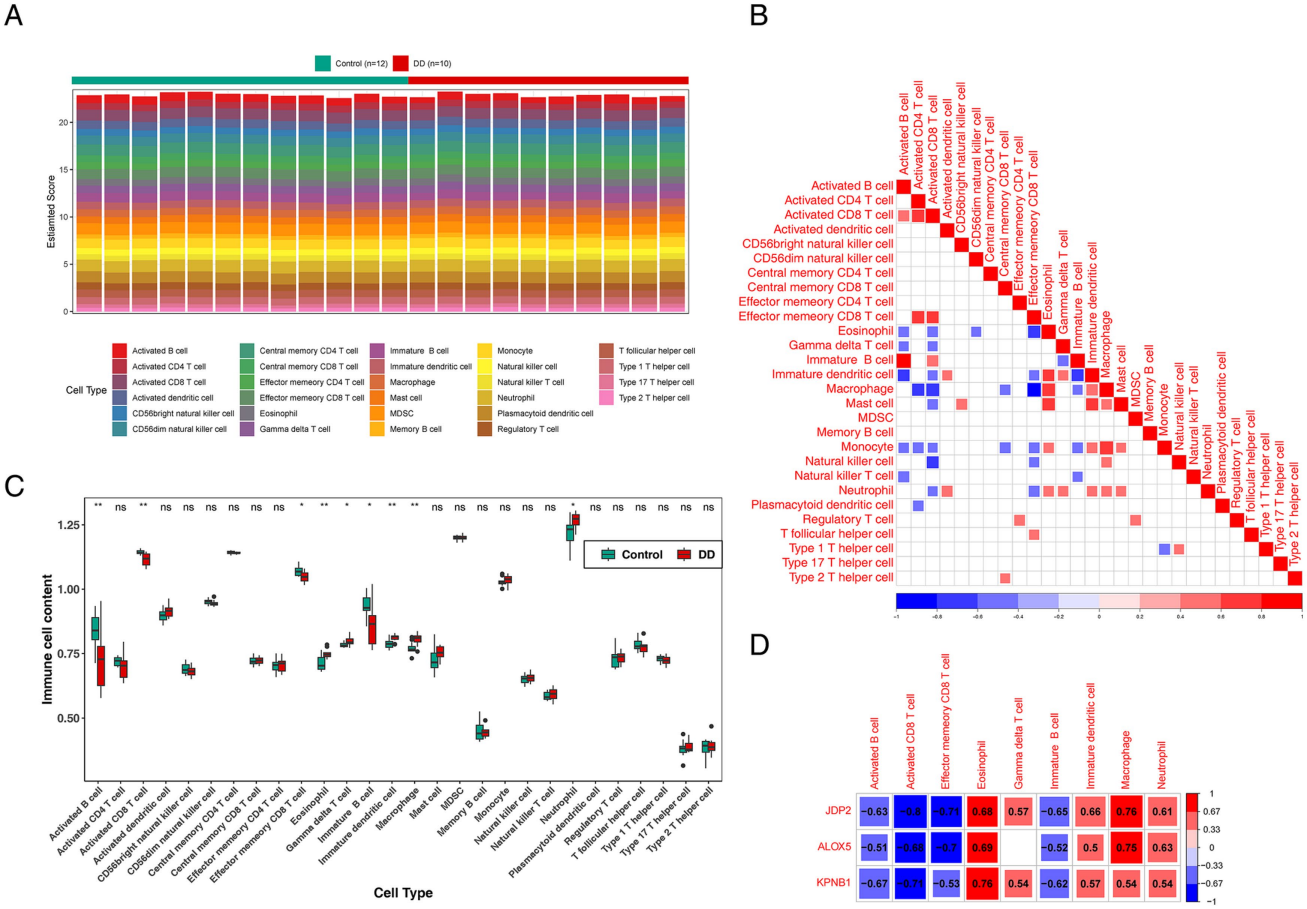
Results of immune cell infiltration analysis in GSE 76826. **(A)** The relative amount of infiltration of 28 distinct immune cell subtypes in the peripheral blood of patients with DD. **(B)** Correlation among 28 different immune cell subgroups; white denotes no association between any given immune cell group, while red and blue show positive and negative correlations, respectively. **(C)** A comparison of 28 different immune cell types, where the normal group is shown in green and the depressive group is shown in red. The treatment group represents depression, and the control group represents the normal group. **(D)** Three hub genes related to histone acetylation and nine immune cells are correlated. The positive association increases with color redder. The negative connection increases with a beautiful blue hue.

To observe the composition of immune cells between the normal group and the DD group samples, We conducted immune infiltration analysis. The distribution proportion and correlation heat map of the 28 immunoinfiltrating cells in GSE76826 are displayed in Figures 5A,B. Nine types of immune cells were identified: eosinophils, gamma delta T cells, activated B cells, activated CD8 T cells, effector memory CD8 T cells, immature B cells, immature dendritic cells, macrophages, and neutrophils (Figure 5C). This suggested that the hub genes might have influenced the level of immune cell infiltration in DD. A substantial negative correlation was identified between *JDP2* and activated CD8 T cells (cor = −0.8), whereas *KPNB1*, eosinophils, and macrophages showed a high positive correlation (cor = 0.76) (Figure 5D).

### 3.5 lncRNA-miRNA-mRNA network construction based on hub genes

**Figure 6:**
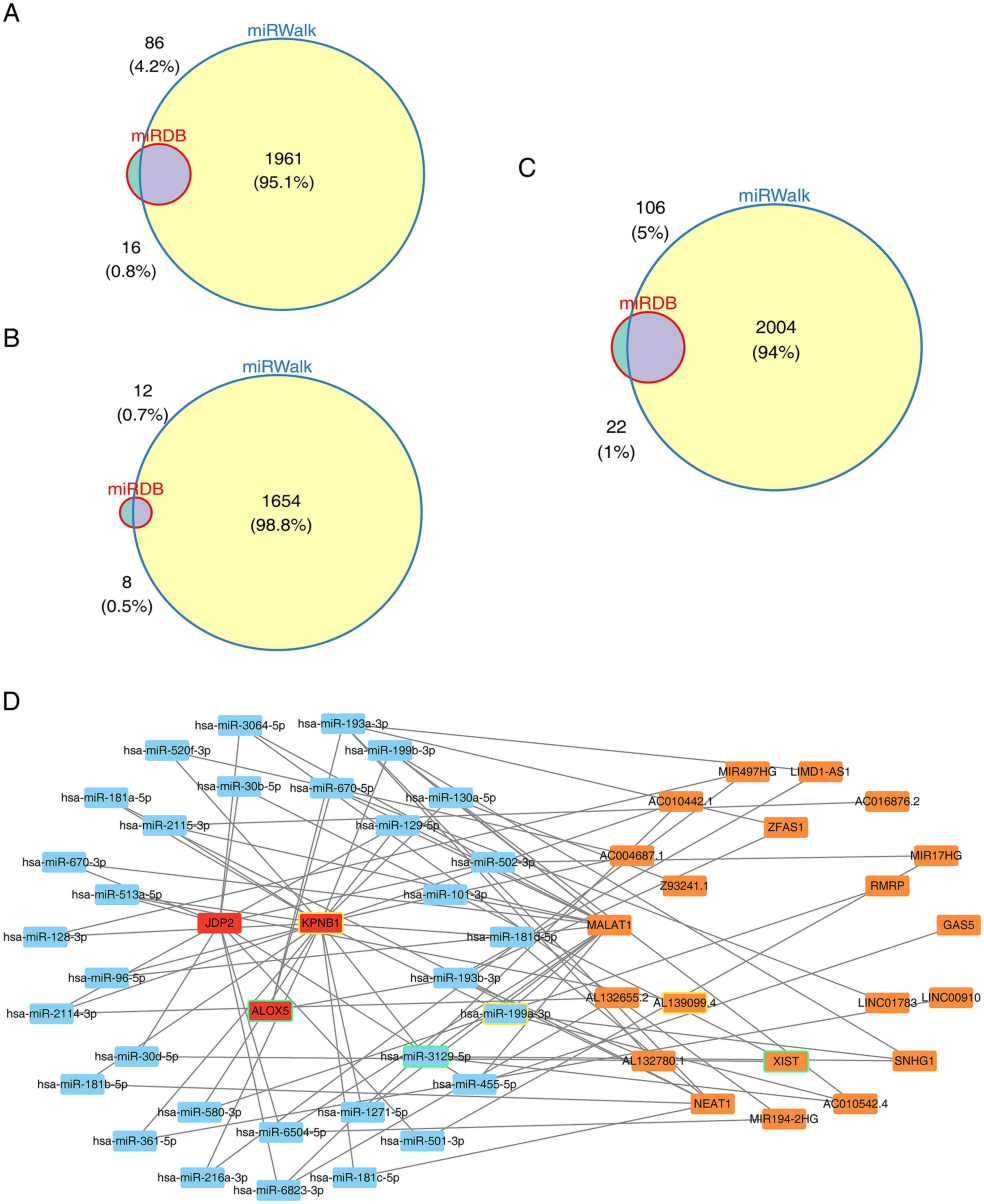
lncRNA-miRNA-mRNA Network construction based on hub genes. **(A–C)** Venn diagrams showing the intersection of miRNA numbers of hub genes from the GSE98793 and GSE76826 databases. **(D)** CeRNA network: regulating gene expression through interactions between RNA molecules, investigation of the lncRNA-miRNA-mRNA network. Orange denotes predicted lncRNA, red indicates hub genes, and blue indicates predicted miRNA.

To gain a deeper understanding of the potential mechanisms of hub gene regulation, we conducted a ceRNA network analysis. Using the two databases, the numbers of miRNAs predicted by *JDP2*, *ALOX5*, and *KPNB1* were 86, 12, and 106, respectively (Figures 6A–C). Based on the common miRNAs, 1,599 lncRNAs were identified. The lncRNA-miRNA-mRNA network contained 56-nodes and 87-edges. For example, XIST-hsa-miR-3129-5p-ALOX5 and AL139099.4-hsa-miR-199a-3p-KPNB1 were among the regulatory relationships identified (Figure 6D). These results demonstrated the interactions between different molecules, helping to reveal the potential mechanisms of hub gene regulation.

### 3.6 Construction of hub gene-drug network based on hub genes

**Figure 7:**
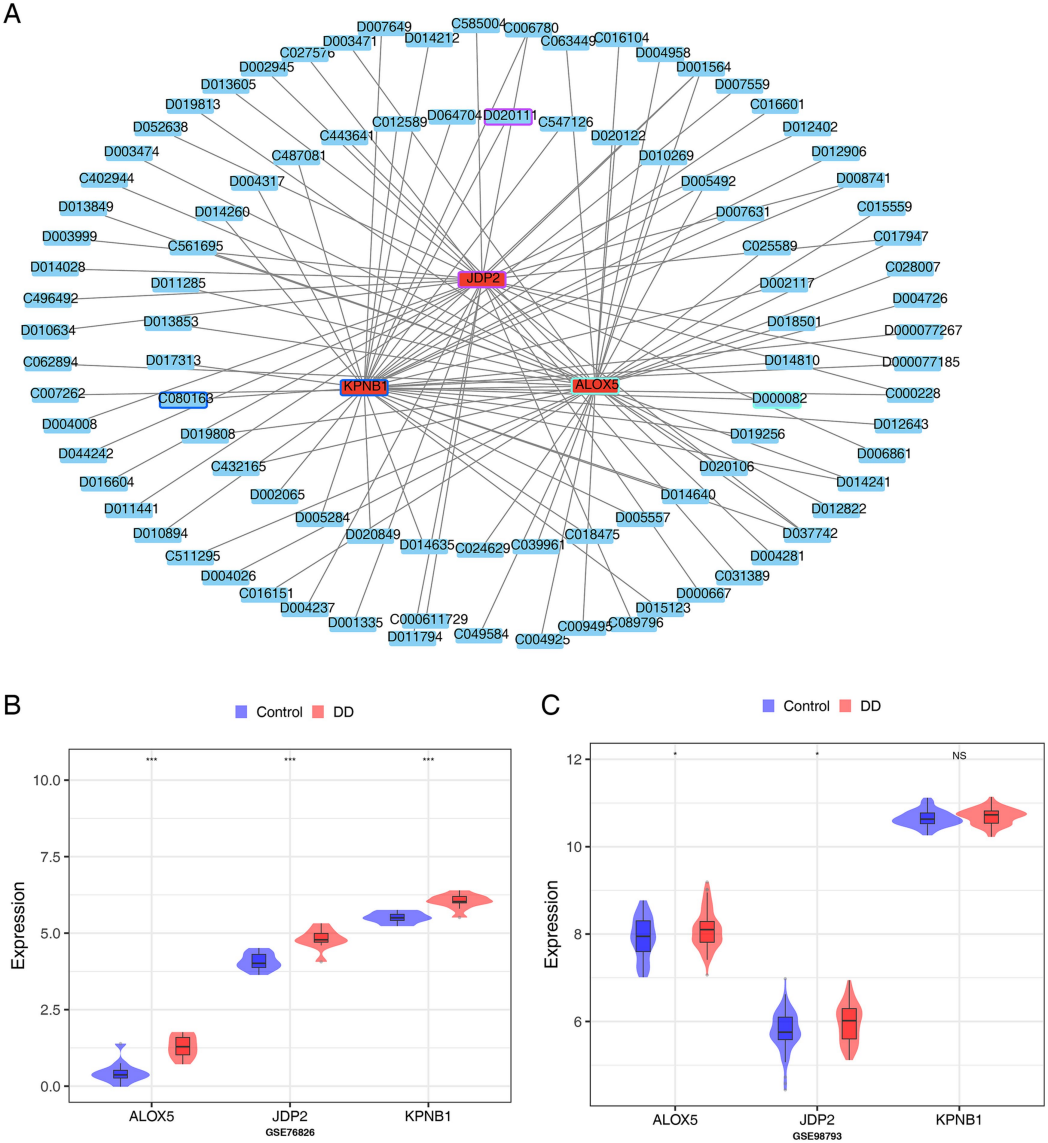
Construction of hub gene-drug Network based on hub genes. **(A)** Hub gene-drug network analysis: the relationship between key genes and inhibitory drugs, red represents hub genes, and blue represents predicted drugs. **(B)** Expression of potential biomarkers in the GSE98793 database. **(C)** Expression of potential biomarkers in the GSE76826 database.

To predict potential drugs related to the hub gene and DD, we conducted a drug prediction analysis. The hub gene-drug network contained 98-nodes and 106-edges. For instance, C080163 suppressed *KPNB1* expression, D000082 suppressed *ALOX5* expression, and D020111 suppressed *JDP2* expression (Figure 7A). Finally, we verified the expression. The expression of *ALOX5*, *JDP2*, and *KPNB1* was lower in the control samples than in the DD samples of GSE76826. In GSE98793, the trends of *ALOX5* and *JDP2* were consistent with those of GSE76826, whereas *KPNB1* did not differ significantly in GSE98793 (Figures 7B,C).

### 3.7 Validation of hub gene expression in clinical samples

**Figure 8:**
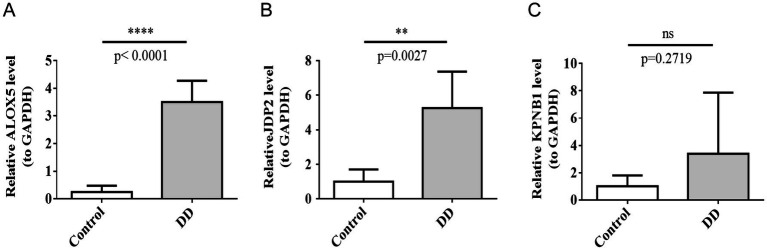
Three hub genes’ expression in clinical samples was validated by qPCR. * indicates *p*-value < 0.05. **(A)** JDP2, **(B)** ALOX5, **(C)** KPNB1.

Blood samples were taken from five DD patients and five healthy controls to verify the expression of hub genes. The reverse transcription quantitative PCR (RT-qPCR) results showed that the relative expression levels of *ALOX5* and *JDP2* were significantly higher in the DD group than those in the control group (Figures 8A,B). However, no significant difference was observed in *KPNB1* expression between the control and DD groups (Figure 8C). These results suggested that the expression levels of ALOX5 and JDP2 might have influenced DD.

## 4 Discussion

DD is influenced by both genetic and environmental factors. Clinical manifestations of DD include cognitive impairment, persistently depressed mood, and a decrease in volitional activity, with severe cases potentially presenting with symptoms such as hallucinations (Liu et al., 2023). The diagnosis of DD remains difficult due to its heterogeneity and complex pathological characteristics. In the absence of objective diagnostic criteria, the current diagnosis is primarily based on clinical evaluation of patients’ self-reported symptoms. The development of DD may be influenced by histone modifications caused by various environmental variables. Among these modifications, histone acetylation plays a crucial role in DD (Montagud-Romero et al., 2016). Studying histone acetylation-related biomarkers could provide deeper insight into the pathogenesis of DD and offer new perspectives on diagnostic and treatment strategies.

In this study, biomarkers associated with DD were screened using LASSO and Boruta machine learning methods. Through ROC curve analysis, *ALOX5*, *JDP2*, and *KPNB1* were identified as the three key genes associated with histone acetylation. *ALOX5* (Arachidonate 5-lipoxygenase) is a non-heme iron-containing dioxygenase involved in leukotriene biosynthesis and regulation of inflammatory responses and various types of cell death (Sun et al., 2019). Ortega et al. found increased expression of *ALOX5* in placental tissues when comparing biomarkers from 22 pregnant women with gestational psychosis and 20 healthy pregnant women. Furthermore, the study discovered that *ALOX5* overexpression was directly associated with abnormally increased glucocorticoid levels in pregnant women experiencing their first psychotic episode (Ortega et al., 2023).

Similarly, a meta-analysis by Hubbard and Miller’s revealed that individuals undergoing their first psychotic episode exhibited abnormally high levels of glucocorticoids in their blood (Hubbard and Miller, 2019). This suggests that *ALOX5* may contribute to the pathological changes associated with DD by regulating the HPA axis. Recent research has shown a direct correlation between inflammation and DD (Sarno et al., 2021), with *ALOX5* possibly serving as a crucial mediator. However, how *ALOX5* contributes to the development and progression of DD remains unclear (Joshi and Praticò, 2013). There exists a significant association between histone acetylation and inflammation. Both inflammatory and anti-inflammatory genes are regulated by histone acetylation, thereby determining their activation status (Daskalaki et al., 2018). Histone acetylation is controlled by histone acetyltransferases (HATs) (Adcock et al., 2005). The acetylation mediated by HATs typically facilitates gene transcription by unraveling compact chromatin structures (Grunstein, 1997). As a crucial member of the HAT family, p300 not only acetylates histones but also functions as an adaptor factor for transcription factors (Eckner et al., 1994). Notably, potential p300 binding sites have been identified on the *ALOX5* promoter (Zhang et al., 2023), and studies suggest that cortisol may regulate *ALOX5* gene expression through p300 (Wang et al., 2012). These findings indicate a close relationship between the *ALOX5* gene and histone acetylation. Therefore, we hypothesize that *ALOX5* might activate inflammatory response systems through histone acetylation-mediated mechanisms, potentially contributing to the pathogenesis of DD.

The *JPD2* (Jun dimerization protein 2), a member of the stress protein family, is recognized as an AP-1 repressor protein involved in chromatin assembly regulation, and both positive and negative transcriptional regulation (Lohoff et al., 2022). In addition to its involvement in several cellular functions, active AP-1 has been connected to the molecular mechanisms underlying a number of illnesses, such as cancer, rheumatoid arthritis, psoriasis, and asthma. *JPD2* has been shown to regulate oxidative stress (Tsai et al., 2016; Wuputra et al., 2022). JDP2 is part of the Nrf2-MafK complex, which is involved in detoxification and antioxidant functions. This is accomplished by triggering several detoxifying or antioxidant enzymes, binding to antioxidant response elements (ARE) *in vivo*, and enhancing ARE-dependent gene expression. These enzymes are essential for defending tissues and cells against endogenous reactive oxygen species (ROS) and external carcinogens (Tanigawa et al., 2013). Several clinical disorders, such as cancer, cardiovascular disease, inflammation, and neurodegeneration, are associated with oxidative stress and ROS. Oxidative stress caused by excessive ROS is one of the contributing factors to DD. Patients with DD exhibit decreased antioxidant capacity and higher markers of oxidative stress. Several studies have demonstrated that ROS significantly inhibit histone acetylation, and histone hypoacetylation is associated with oncogenic potential and cytotoxicity (Kang et al., 2003). Furthermore, histone acetylation has been implicated in both the pathophysiology and treatment of depression (Zhu et al., 2021). *JDP2* suppresses HAT activity through its interaction with histones and the binding of its basic bZIP domain to DNA. Although the histone-binding region of *JDP2* lacks typical acidic residues, it remains critical for HAT inhibition, with the basic region in the bZIP domain also playing a significant role. *JDP2* can inhibit p300-mediated acetylation even in the presence of excess histones (Jin et al., 2006). We therefore hypothesize that *JDP2* may influence histone acetylation by modulating ROS levels, thereby playing a pivotal role in regulating depression-related genes.

*KPNB1* is a key protein in the nuclear transport protein β family, responsible for mediating the transport of proteins from the cytoplasm to the nucleus. It comprises 19 tandem HEAT repeat sequences. It has been reported that *KPNB1* plays a pivotal role in epigenetic regulation, particularly in gene silencing and gene expression modulation (Dong et al., 2018). Studies have revealed that elevated expression of *KPNB1* is associated with poor patient prognosis. Notably, DD1 combined with *KPNB1* inhibitors effectively suppresses gastric cancer cell proliferation and tumor growth by enhancing both genomic and non-genomic activities of Nur77, suggesting *KPNB1* as a promising therapeutic target in cancer treatment (Zhang et al., 2024). Furthermore, *KPNB1* has been shown to interact with immobilized H4 (histone), while TNPO1 (KPNB2, MIP1), an importin closely related to *KPNB1*, interacts with the H3 tail. These findings collectively underscore the potential biological significance of KPNB1 in cellular processes (Apta-Smith et al., 2018). However, there is currently limited research on whether the *KPNB1* gene can serve as a specific diagnostic biomarker for mental illnesses to further investigate related therapeutic targets, especially for DD. The present study confirms that these three biomarkers were closely associated with the diagnosis and treatment of DD. However, further investigation is required to elucidate their mechanisms of action.

The present study identified three biomarkers that co-enriched in ribosomes, fc-gamma-r-mediated phagocytosis, and olfactory transduction pathways. Ribosomal dysregulation is a common feature of depression in humans and chronic stress in mice (Zhang et al., 2023). Previous studies have demonstrated that anomalies in the immune and inflammatory systems are linked to the pathophysiology of DD (Richardson et al., 2022), with fc-gamma-r-mediated-phagocytosis being one of the pathways involved. This pathway stimulates phagocytosis, which subsequently increases phospholipase D (PLD) activity. This, in turn, induces phosphatidic acid (PA) production in the plasma membrane of macrophages and promotes phagosome formation (Ji et al., 2016). Furthermore, a lack of olfactory stimulation was significantly correlated with DD. The olfactory and emotional processing pathways share a common anatomical foundation (Croy and Hummel, 2017), and the extent of olfactory impairment can serve as an indicator of depression severity. Additionally, the loss of olfaction may increase the likelihood of developing DD (Leon and Woo, 2022).

Studies have linked DD to leukocytosis, an increased neutrophil-to-lymphocyte ratio, and an elevated CD4 + to CD8 + T-cell ratio (Mazza et al., 2018). It is also associated with systemic immunological activation (Mazza et al., 2018). Through the analysis of DD immune cell infiltration, we discovered that the DD group exhibited higher neutrophil infiltration than the control group, consistent with the observations by Lynall et al. (2020). Their study, which analyzed peripheral blood samples from 206 patients with DD and 77 healthy controls, identified 14 immune cell subtypes and reported an increase in neutrophil numbers in patients with DD; this increase was positively correlated with DD symptom scores, predicting changes in symptom severity (Lynall et al., 2020). Furthermore, a previous study found that the serum of patients with DD had higher levels of several immune cells, including monocytes, dendritic cells, and macrophages (Beurel et al., 2020), which is consistent with our findings. Additionally, DD has been associated with immune suppression characterized by a reduced lymphocyte proliferation response or a decreased T helper cell count (Schleifer et al., 1989). CD8 + T cells play a crucial role in immune regulation, and previous studies have suggested that modulating the immunological microenvironment in depressed mice is possible by decreasing CD8 + T cell apoptosis (Lu et al., 2017). Additionally, Magioncalda et al. reported a strong correlation between bipolar disorder and decreased circulating CD8 + T cell counts (Magioncalda et al., 2018). an important regulator of CD8 + T cell depletion during long-term viral infections and malignancies has been found to be AP-1 (Magioncalda et al., 2018). In our study, activated CD8 + T cells exhibited a negative correlation with *JDP2,* and lower levels of activated CD8 + T cell infiltration were observed, which is consistent with previous studies.

Collectively, the pathogenesis and pathophysiology of DD are significantly influenced by neuroimmune system interactions. In cells where *KPNB1* was inhibited, the transcriptional activities of AP-1 and NFκB, critical for cancer cell biology and the expression of inflammatory target genes, were repressed (Papavassiliou and Musti, 2020). Concomitantly, the expression of interleukin-6, interleukin-1 β, tumor necrosis factor α, and target genes of granulocyte colony-stimulating factor, NFkB, and AP-1 was found to be markedly diminished in experiments employing nuclear input inhibition of *KPNB1*. *KPNB1* has been shown to suppress these transcription factors’ activities, making cancer cells more invasive (Stelma and Leaner, 2017). Studies have also found that KPNB1 expression is up-regulated in a number of cancers (Cai et al., 2024), and dysregulation of KPNB1 is closely linked to carcinogenesis. Moreover, *ALOX5* regulates T cell pyroptosis in rheumatoid arthritis. Arachidonic acid (AA)-regulated Ca2 + −selective (ARC) channels promote CD 4 + T cell pyroptosis and elevate the expression of *ALOX5* in rheumatoid arthritis CD 4 + T cells (Cai et al., 2024). *ALOX5* has also been reported to alter neuronal function, which may explain why mice lacking *ALOX5* display increased defensive behaviors against anxiety (Joshi and Praticò, 2011). Moreover, the placental tissues of pregnant women experiencing their first psychotic episode showed higher expression of ALOX5 compared to healthy control (Zhang et al., 2023). However, whether the interactions between these genes and immune cells contribute to the progression of DD remains to be determined and requires further investigation.

In summary, through bioinformatics and machine learning approaches, we have identified histone acetylation-related biomarkers *ALOX5*, *JDP2*, and *KPNB1* associated with depression. These genes hold significant clinical translational potential in depression research. With in-depth understanding of their mechanistic roles in depression pathogenesis, these genes may emerge as novel biomarkers, providing new perspectives for early diagnosis, pathological monitoring, and personalized treatment of DD. Specifically, *ALOX5* might activate the inflammatory response system through histone acetylation mechanisms, thereby triggering DD; *JDP2* could regulate ROS levels to influence histone acetylation, playing a pivotal role in controlling depression-related gene expression and offering guidance for individualized therapy; while *KPNB1* may serve as a crucial mediator in neurotransmission and intracellular signaling pathways, facilitating assessment of disease progression and therapeutic efficacy in DD. However, this study has certain limitations. Firstly, the analysis based on the dataset may have certain biases in terms of sample sources and clinical background. Many *in vivo* and *in vitro* variations in environmental factors and intercellular interactions cannot be predicted solely through bioinformatics analysis. Secondly, DD exhibits clinical heterogeneity. This study did not comprehensively document the specific disease subtypes of enrolled patients. While we employed the ssGSEA method to estimate immune cell infiltration in depression patients, these predictive results require further validation through additional research. Therefore, subsequent studies should incorporate samples from more diverse backgrounds and enhance validation through *in vivo* and *in vitro* experiments. Although genes JPD2, ALOX5, and KPNB1 have demonstrated significant potential in basic research - potentially playing crucial roles in early diagnosis, prognostic evaluation, treatment response monitoring for cancer, immune and inflammatory diseases, and serving as key targets for precision therapy - their mechanistic roles in depression warrant further investigation. Clinical translation still requires additional validation, particularly through more *in vivo*/*in vitro* experiments and exploration of targeted therapeutic strategies, to develop more effective treatment regimens for patients. Despite existing limitations, this study provides valuable references for the clinical diagnosis and treatment of DD.

## 5 Conclusion

Using a series of bioinformatics methods, this study identified three key genes (*ALOX5*, *JDP2*, *KPNB1*) that play significant roles in DD. The findings revealed that these genes were highly correlated with immune cell infiltration levels and had a superior diagnostic performance. These genes have undergone extensive validation and may have practical implications for the diagnosis and treatment of DD. In addition, these genes may play a role in the immunological, inflammatory, or signaling pathways that contribute to the pathophysiology of DD. This insight could open new avenues for research and help uncover novel biomarkers and therapeutic targets for DD.

## Data Availability

The original contributions presented in the study are included in the article/supplementary material, further inquiries can be directed to the corresponding author.

## Footnotes

^1^ https://www.ncbi.nlm.nih.gov/

^2^ https://www.genecards.org/

^3^ http://www.mirdb.org

^4^ http://mirwalk.umm.uni-heidelberg.de/

^5^ http://starbase.sysu.edu.cn

^6^ http://ctdbase.org/

^7^ http://ctdbase.org/
